# New Method for 5′−Nucleotidase Preparation and Evaluation of Its Catalytic Activity

**DOI:** 10.3390/foods13050708

**Published:** 2024-02-26

**Authors:** Yin Zhang, Qing Zeng, Yingjie Zhang, Pengcheng Zhang, Qing Li, Jiao Zhou, Li Dong, Zhongli Pan

**Affiliations:** 1 Meat Processing Key Laboratory of Sichuan Province, Chengdu University, Chengdu 610106, China; 2 Department of Biological and Agricultural Engineering, University of California, Davis, One Shields Avenue, Davis, CA 95616, USA; zlpan@ucdavis.edu

**Keywords:** pork, umami, 5′−nucleotidase, isolation and purification

## Abstract

In this study, we established a new methodology for preparing 5′−nucleotidase (5′−NT) with the aim of enhancing our understanding of its enzyme activity and laying a basis for regulating the content of umami−enhancing nucleotides in pork. 5′−NT was prepared with Sephadex gel filtration and reverse−phase high−performance liquid chromatography, and its enzymatic properties and catalytic activity were evaluated. The results show that the molecular weight of the prepared 5′−NT was 57 kDa, the optimal catalytic temperature was 40 °C, and the optimal pH was 8. Zn^2+^, and sucrose showed inhibitory effects on the activity of 5′−NT, while K^+^, Na^+^, Ca^2+^, Mg^2+^, glucose, fructose, and trehalose promoted the activity of the studied compound. The prepared 5′−NT exhibited higher catalytic activity and selectivity against IMP compared with its commercial counterpart, while its catalytic activity against XMP was not significant (*p* > 0.05). In brief, we established a new methodology for preparing 5′−NT, enhancing our understanding of its enzyme activity and providing a solid basis for regulating the content of umami−enhancing nucleotides in pork through the control of endogenous 5′−NT activity.

## 1. Introduction

China has consistently ranked first in the world in meat production for many years [1]. Pork, which is mainly stored and sold in a chilled state, accounts for about 50% of consumed meat [2,3]. To enhance the umami or meaty flavor of pork, umami−enhancing nucleotides are used together with glutamate, as the use of these substances alone does not achieve the same effect [4,5]. Therefore, their content is considered a key quality index in stored pork [2,6]. The main umami−enhancing nucleotides in pork include adenosine monophosphate (AMP), guanylic acid (GMP), cytidine monophosphate (CMP), inosine monophosphate (IMP), xanthosine monophosphate (XMP), and uridylic acid (UMP) [6,7]. Recent investigations have found that the content of these nucleotides in postmortem pork meat decreases with the increase in storage time and that the main reason for this is the activity of endogenous 5′−nucleotidase (5′−NT; EC:3.1.3.5) in pork [2].

5′−NT is a phosphate ester hydrolase that is commonly present on the cytoplasmic membrane of various tissues, such as muscles [8]. It can specifically catalyze the hydrolysis of nucleotides, hydrolyzing the phosphate bound to the 5′ position of pentose; by virtue of this, it can degrade IMP [9], a key umami−enhancing nucleotide. The content of IMP in pork is high [3], and the decrease in its content greatly abates the taste of meat [6]. One of the methods to inhibit the decomposition of IMP and other umami−enhancing nucleotides in pork during chilled storage is suppressing the activity of 5′−NT. However, the market availability of 5′−NT is very limited.

The references reported about preparation of 5′−NT mainly include extraction and gene expression. The materials used to extract 5′−NT are pig intestines [10]; plasma membranes from mouse liver [11], pig lymphocytes [12] and human liver [13]; cod (*Gadus microcephalus*) [14] and carp *Cyprinus carpio* [15] muscle; *Trimeresurus albolabris* venom [16]; bovine brain [17]; and *Escherichia coli* K−372 cells [18]. The typical procedures are as follows: fragmentation with oscillation shock or sonic disruption, rinsing with a buffer (pH 6.8~8.4), and centrifugation. The supernatant is mixed with salt and centrifuged (filtrated), and then purified with Sephadex gel. The gene−expression method of preparing 5′−NT involves expressing the 5′−NT gene in *Pichia pastoris* GS115 [19] and *Escherichia coli* BL21 [20]. The expressed protein is decomposed and purified with the extraction method to obtain 5′−NT. Nowadays, the 5′−NT sold on the market is mainly extracted from microorganisms. The aims of this investigation were to establish a new methodology for preparing 5′−NT and to study the enzyme activity of this compound. The main areas investigated included 5′−NT extraction and purification, −the properties of 5′−NT, a hydrolysis effect comparison, and the effect of 5′−NT on nucleotides in chilled pork.

## 2. Materials and Methods

### 2.1. Materials

Methanol and acetonitrile (of chromatographic grade) were purchased from Thermo Fisher Scientific (China) Co., Ltd. (Shanghai, China). Chromatographic standards of AMP, GMP, CMP, IMP, XMP, UMP, hypoxanthine riboside (HxR), and hypoxanthine (Hx) were purchased from Sigma Company (Saint Louis, MO, USA). Electrophoretic reagents were purchased from Shanghai Yuanye Biotechnology Co., Ltd. (Shanghai, China). Other chemicals, all of analytical grade, were purchased from Chengdu Cologne Chemical Co., Ltd. (Chengdu, China).

### 2.2. Crude 5′−NT Extraction

Crude 5′−NT was extracted according to the method described by Jin, et al. [21] with some modifications. White pork tenderloin in an amount of 5000 g was obtained from five male hybrid white pigs (Meishan pig × Chenghua pig × Landrace pig; one year old, weight of 75 ± 5 kg) subject to the same feeding conditions [2]. The samples were obtained on the day the pigs were slaughtered. The pork tenderloin was washed, and the fascia was removed; then, the samples were minced using a TJ−12 meat grinder (Zhucheng Jiaxin Food Machinery Co., Ltd., Zhucheng, China) and placed into a sterile homogenization bag. The minced meat and pre−cooled Tris−HCl buffer (0.05 mol/L, pH 7.5) were mixed in a ratio of 3:1 (*v*:*w*). A SCIENTZ−11 sterile homogenizer (Ningbo Xinzhi Biotechnology Co., Ltd., Ningbo, China) was used to homogenize the mixture for 2 min; then, the mixture was allowed to stand at 4 °C for 1 h. The supernatant was mixed with pre−cooled n−butanol 0.2 times in volume at 4 °C for 8 h to remove the fat and then centrifuged using a TGL−1650 apparatus at 12,000 rpm and 4 °C for 20 min (Sichuan Shuke Instrument Co., Ltd., Chengdu, China). The supernatant was vacuum−filtered with slow filter paper and allowed to stand for layers to separate. The layer at the bottom was the crude 5′−NT solution.

### 2.3. Ammonium Sulfate Precipitation

The crude 5′−NT solution was precipitated according to the method described by Yao [22]. Ammonium sulfate was slowly added into the crude 5′−NT solution to a saturation level of 0.35. After complete dissolution, the mixture was allowed to stand at 4 °C for 4 h and was successively centrifuged at 12,000 rpm for 20 min to obtain the supernatant. The latter was again supplemented with ammonium sulfate to the saturation level of 0.65 using the same procedure, and the mixture was allowed to stand at 4 °C for 4 h. After centrifugation at 12,000 rpm for 20 min, the solid−phase solution was separated and dissolved in 0.05 mol/L Tris−HCl buffer (pH 7.5).

### 2.4. Sephadex Gel Chromatography Separation

G−75 dextran gel was used to purify the precipitated 5′−NT. Tris−HCl buffer (pH 7.5) was used as the mobile phase. Prior to separation, 5 mL of the precipitated 5′−NT solution was filtered with a 0.45 μm membrane (Shanghai Xinya purification equipment Co., Ltd., Shanghai, China), and the filtrate was injected into a G−75 chromatography column system (HD−300 UV detector and HL−2B constant−flow pump). The required elution fractions were collected.

### 2.5. Reverse−Phase High−Performance Liquid Chromatography (RP−HPLC) Purification

To establish the purification conditions, the elution fractions were loaded onto a Wukong K2025 RP−HPLC apparatus (Haineng Future Technology Group Co., Ltd., Shanghai, China) with a column of SinoChrom 300Å ODS−AP C18 (4.6 mm × 250 mm × 5 μm, Dalian Yilite Analytical Instrument Co., Ltd., Dalian, China). The established chromatographic conditions were as follows: The mobile phase was acetonitrile solution containing 1% TFA and 1% TFA, with an injection volume of 90 μL, an elution time of 25 min, a flow rate of 1 mL/min, and a detection wavelength of 220 nm. For gradient elution, 1% TFA solution and deionized water were maintained in a volume ratio of 98:2 for 5 min, in a volume ratio of 50:50 for 5 min, in a volume ratio of 10:90 for 10 min, and finally, in a volume ratio of 98:2 for 5 min.

According to the established chromatographic conditions, 5′−NT was prepared using an Agilent 1260 preparative liquid chromatography apparatus equipped with diode array detectors (Agilent Technology (China) Co., Ltd., Beijing, China) and a column of SinoChrom 300Å ODS−AP C18 (10 mm × 250 mm × 5 μm, Dalian Yilite Analytical Instrument Co., Ltd., Dalian, China). A sample of 425 μL in volume was loaded and separated at a flow rate of 4.7 mL/min to obtain purified 5′−NT. A diode array detector was used for full−band scanning during elution, and the other parameters were the same as those of the analysis conditions above.

### 2.6. Purity Verification

We verified the purity of the obtained 5′−NT by comparing it with commercial 5′−NT (extracted from microorganisms; purchased from Shanghai Yuanye Biotechnology Co., Ltd., Shanghai, China) with RP−HPLC and denatured gel electrophoresis. The RP−HPLC conditions were the same as those described in Section 2.5; ultrapure water was the blank control, and commercial 5′−NT was the positive control. SDS−PAGE was performed according to the method described by Zhang, et al. [23] with slight modifications. Briefly, 1 mg/mL of purified 5′−NT solution and 1 mg/mL of commercial 5′−NT solution were independently mixed with the sample buffer (1 mol/L Tris–HCl (pH 6.8) containing SDS [10 g/100 mL], glycerol [50 g/100 mL], β−ME [10 g/100 mL], and 1% [1 g/100 mL] bromophenol blue) in a 4:1 (*v*/*v*) ratio. Then, 10 μL samples were loaded into polyacrylamide gel made of 10% running gel and 5% stacking gel and were subjected to electrophoresis at 15 mA concentrated current and 30 mA separate current. After separation, the gel was stained with 0.02 g/100 mL Coomassie Brilliant Blue R−250 (Shanghai Bio Life Science & Technology Co., Ltd., Shanghai, China) in methanol (50 mL/100 mL) and acetic acid (7.5 mL/100 mL), destained with methanol (50 mL/100 mL) and acetic acid (7.5 mL/100 mL), and re−destained with methanol (5 mL/100 mL) and acetic acid (7.5 mL/100 mL). The electrophoresis gel was analyzed with a Gel Pro Analyzer 4.0, and the molecular weight of the commercial and purified 5′−NT was calculated according to the method described by Zhang, et al. [24].

### 2.7. Optimal pH Determination

The optimal pH of the purified 5′−NT was determined according to the method described by Zhang [25] with slight modifications. The highest 5′−NT activity was taken as 100%, and the relative activity of 5′−NT at different pH levels was calculated with reference to 100% [25]. The pH levels (4.0, 5.0, 6.0, 7.0, 8.0, 9.0, 10.0, and 11.0) were adjusted with pH buffer solutions, which included 20 mmol/L acetic acid sodium acetate buffer (pH 4.0~5.0), 20 mmol/L potassium dihydrogen phosphate sodium hydroxide buffer (pH 6.0~7.0), 50 mmol/L Tris hydrochloric acid buffer (pH 8.0~9.0), and 50 mmol/L sodium bicarbonate sodium hydroxide buffer (pH 10.0~11.0).

### 2.8. Optimal Temperature Determination

One milliliter of 5′−NT solution was placed in an SPL−150 biochemical incubator (Tianjin Lebo Terui Instrument Equipment Co., Ltd., Tianjin, China) and incubated at 20 °C, 25 °C, 30 °C, 35 °C, 40 °C, 45 °C, 50 °C, 55 °C, 60 °C, 65 °C, and 70 °C for 30 min, respectively. The relative activity of 5′−NT at different temperatures was calculated according to the method described by Zhang [25].

### 2.9. Effect of Salt Ions on 5′−NT Activity

5′−NT solution of 1 mL in volume was mixed with 1 mL of NaCl, KCl, CaCl_2_, MgCl_2_, or ZnCl_2_ solution, whose concentration was adjusted to 0.5 mmol/L, 1 mmol/L, 1.5 mmol/L, 2 mmol/L, 2.5 mmol/L, and 3 mmol/L in different experiments. We used 5′−NT solution containing 1 mL of ultrapure water as the control. The relative activity of 5′−NT with different salt ions was calculated according to the method described by Zhang [25].

### 2.10. Effect of Carbohydrates on 5′−NT Enzyme Activity

5′−NT solution of 1 mL in volume was mixed with 1 mL of sucrose, trehalose, glucose, or fructose solution, whose carbohydrate concentration (*m*:*v*) was adjusted to 5%, 10%, 15%, 20%, 25%, and 30% in different experiments. We used 5′−NT solution containing 1 mL of ultrapure water as the control. The relative activity of 5′−NT with different carbohydrates was calculated according to the method described by Zhang [25].

### 2.11. Activity of 5′−NT

The activity of 5′−NT in the supernatant was determined according to Zhang, Y. et al. [2] with slight modifications. 200 μL of the supernatant was loaded into a 5′−NT activity test kit (Nanjing Jiancheng Bioengineering Institute, Nanjing, China), and the activity units were expressed as U⋅g protein^−1^.

### 2.12. Determination of Nucleotides

The nucleotides (GMP, IMP, XMP, AMP, Hx, HxR) in samples were determined according to Zhang, Y. et al. [2] with some modifications. An Agilent 1100 high performance liquid chromatograph (HPLC, Agilent Company, Santa Clara, CA, USA) equipped with Hypersil ODS2−C18 (4.6 mm × 250 mm, 5.0 μm) and an ultraviolet (UV) detector was used. A total of 20 g of the crushed muscle powder was placed in a 50 mL plastic centrifuge tube and mixed with 40 mL of 5% trichloroacetic acid (TCA) solution. The mixture was placed in a 4 °C refrigerator for 2 h before it was centrifuged at the speed of 2725× *g* for 10 min. The supernatant was transferred into a 100 mL beaker and its pH was adjusted to 6.5 with 3 mol/l KOH solution. The solution was filtered with a qualitative filter paper (Fushun Mingzheng Filter Paper Factory, Anhui, China) and diluted to 50 mL with deionized water. The diluted solution was filtered with a 0.45 μm organic membrane (Shanghai Xinya Purification Equipment Co., Ltd., Shanghai, China) and 10 μL of it was injected into the Agilent 1100 HPLC. The optimized gradient chromatographic conditions were as follows: mobile phase A (pure methanol) 2–15%, B (KH_2_PO_4_ buffer (98–85%)) is 0.05 mol/L; column temperature: 30 °C; flow rate: 0.8 mL/min. The nucleotide contents were calculated using the external standard method.

### 2.13. Catalytic Activity Validation

To test the catalytic activity and substrate specificity of the prepared 5′−NT, the nucleotide standards were mixed and used as substrate. A total of 8 mg of GMP, IMP, XMP, or AMP was mixed with 4 mg of HxR and 2 mg of Hx. Each mixture was dissolved into 10 mL of ultrapure water, sonicated for about 5 min, and then divided in two fractions. Volumes of 1 mL of the purified 5′−NT and 1 mL of the commercial 5′−NT were independently added to the nucleotide mixtures, respectively. After mixing, we collected 1 mL from each sample to determine the nucleotide content, which was taken as the pre−reaction value. We incubated the mixtures at 40 °C for 30 min and then again determined the nucleotide content, which was taken as the post−reaction value.

### 2.14. Effect of 5′−NT on Nucleotides in Chilled Pork

To investigate the effect of 5′−NT on nucleotides in chilled pork, one kilogram of white pork tenderloin was used. The meat was washed, and fascia fat removed. The tenderloin tissue was cut into 3 cm × 3 cm ×1 cm slices with the same mass, which were divided into two groups, namely, the control group and the enzyme−treated group. The control group was tumbled with 3 mL of ultrapure water, while the enzyme−treated group was tumbled with 3 mL of purified 5′−NT solution (1 mg/mL). The tumbled samples were stored at 4 °C for 8 h, 24 h, 48 h, 72 h, 120 h, and 144 h. The nucleotide content and 5′−NT enzyme activity were determined according to the methods described by Zhang, Y. et al. [2].

### 2.15. Statistical Analysis

The results were analyzed using ANOVA, with a significance level of 5% (H0: *p* < 0.05). The comparison of the means was performed using Fisher’s LSD tests with the SAS 9.0 statistical package. All the tests were conducted in triplicate.

## 3. Results and Discussion

### 3.1. Separation and Purification of 5′−NT

#### 3.1.1. Sephadex Gel Separation

**Figure 1:**
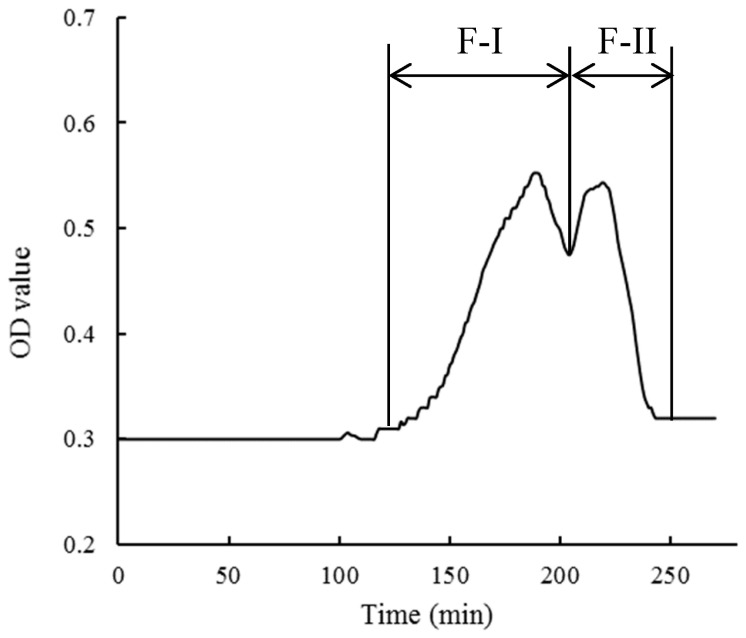
Sephadex G−75 gel separation of the crude 5′−NT. OD: Optical Density; F−I represents Fraction I; F−II represents Fraction II.

The results of the Sephadex G−75 gel separation of crude 5′−NT are shown in Figure 1. The data in this figure indicate that two fractions were obtained from crude 5′−NT, where fraction I (F−I) was obtained in the retention time window from 140 min to 205 min, and fraction II (F−II) in that from 205 min to 245 min. The total enzyme activity, protein content, and specific enzyme activity were 18.35 ± 0.33 U/L, 2.67 ± 0.09 g/L, and 6.87 ± 0.28 U/g protein (Table 1), respectively, in F−I, and 3.82 ± 0.15 U/L, 1.98 ± 0.05 g/L, and 1.92 ± 0.06 U/g protein, respectively, in F−II. Both the total enzyme activity and specific enzyme activity in F−I were significantly (*p* < 0.05) higher than those in F−II. Therefore, F−I was collected to be further separated and purified.

According to previously published research results, the molecular weight of 5′−NT isolated from *Escherichia coli* is 61 kDa [26]; that of purified 5′−NT isolated from black rockfish (*Sebastes inermis*) muscle is 67 kDa [8]; that of 5′−NT isolated from the venom of *Trimeresurus albolabris* is 48.03 kDa [27]; that of 5′−NT isolated from the venom of *Agkistrodon blomhoffii Ussurensis* is 70 kDa [25]. These findings indicate that the molecular weight of most 5′−NT types is in the range of 30–80 kDa. G−75 gel can separate proteins with molecular weights between 3 kDa and 70 kDa. Therefore, it was inferred that the protein separated here using G−75 gel contained 5′−NT. In order to further purify the compound, RP−HPLC was used to separate fraction F−I.

#### 3.1.2. RP−HPLC Separation Results

**Figure 2:**
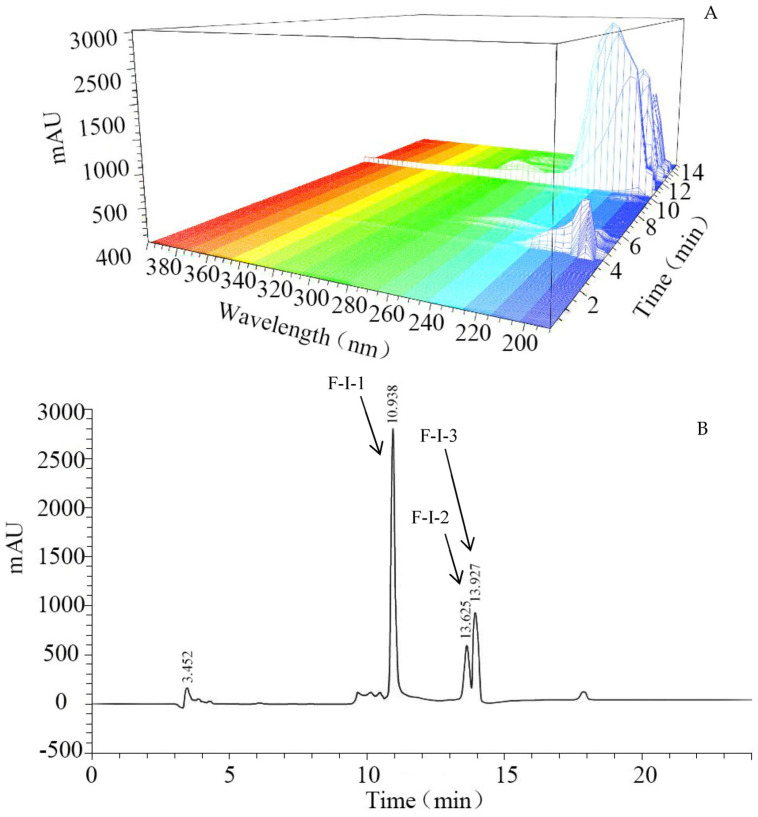
UV full−−band diode array detector (DAD) scanning and RP−−HPLC chromatogram of 5′−−NT. (**A**) UV full−band DAD scanning of 5′−NT; different colored stripes represent different absorbance regions; (**B**) RP−HPLC chromatogram of 5′−−NT.

In order to determine the characteristic absorption wavelength of 5′−NT, fraction F−I was scanned with a diode array detector (DAD, Figure 2A). The signal values reported in Figure 2A indicate obvious absorption peaks for 5′−NT at wavelengths between 190–250 nm and 265–295 nm, the highest absorption intensity being recorded at 220 nm. Therefore, 220 nm was chosen as the characteristic absorption wavelength for 5′−NT. Based on this wavelength, 5′−NT was separated and purified using RP−HPLC (Figure 2B). The results in Figure 2B indicate that there were three sub−fractions in F−I. The specific enzyme activities in sub−fractions F−I−1, F−I−2, and F−I−3 were measured to be 41.19 ± 1.98 U/g protein, 5.39 ± 0.28 U/g protein, and 5.21 ± 0.32 U/g protein (Table 1), respectively. The specific enzyme activity in F−I−1 was significantly (*p* < 0.05) higher than those in F−I−2 and F−I−3. Therefore, fraction F−I−1 was collected through enriching and freeze−drying processes and further used as purified 5′−NT.

It is known that 220 nm is the characteristic absorption wavelength of most proteins, and 5′−NT is a type of protein [26]. Therefore, the maximum absorption of 5′−NT at 220 nm (Figure 2A) suggested that the obtained compound was the target product. The enzyme activity in F−I−1 (41.19 ± 1.98 U/g protein) was significantly (*p* < 0.05) higher than that in F−I−2 or F−I−3 and was found to be 0.00083 times that of the commercial 5′−NT considered in this study (50 U/mg protein). The yield of the 5′−NT was about 1.67 mg/100 g meat. The activity of F−I−1 was higher than that of 5′−NT extracted from cod muscle using TX−100 (1.56 U/mg protein) and Concanavalin A−Sepharose (25.8 U/mg protein) [14], but lower than that of 5′−NT extracted from cod muscle using 5′−AMP Sepharose (828 U/mg protein) [14], or carp muscle using Concanavalin A−Sepharose (65.1 U/mg protein) and 5′−AMP Sepharose 4B (929 U/mg protein) [15]. This might be due to the presence of subunits or polymeric structures attached to the endogenous 5′−NT extracted from the animal tissues [8,16], which affected the activity of the purified 5′−NT. Other investigations also reported the activities of commercial enzymes being higher than those of endogenous proteases; for example, in a previous study, commercial protease showed higher hydrolysis activity than endogenous proteases in hydrolyzing whole anchovy sprat (*Clupeonella engrauliformis*) protein [28].

#### 3.1.3. Purity Comparison

**Figure 3:**
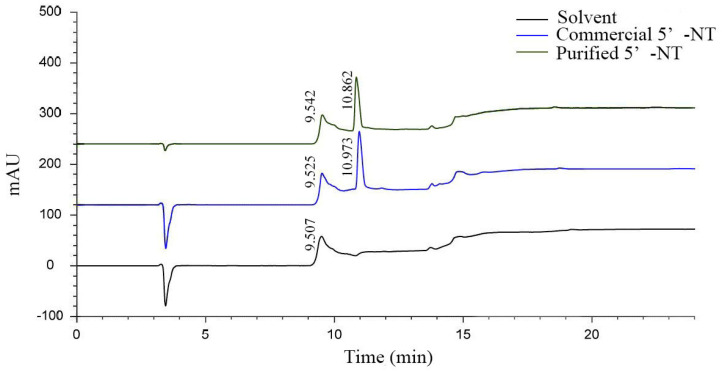
RP−HPLC chromatograms of the commercial and purified 5′−NT.

In order to verify the purity of the prepared 5′−NT, the purified compound was compared with the selected commercial 5′−NT using RE−HPLC (Figure 3). The results in Figure 3 show that the purified and commercial 5′−NT had positive peaks at 10.862 min and 10.973 min, respectively. The relative retention time of the former (10.863 − 9.542 = 1.32) was shorter than that of the latter (10.973 − 9.525 = 1.448). According to the principle of chromatographic separation, it can be inferred that the molecular weight of the purified 5′−NT is likely to be smaller than that of the commercial counterpart. To confirm this speculation, the molecular weights of both compounds were measured with SDS−PAGE (Section 3.1.4).

The source of 5′−NT has a significant impact on its molecular weight. For example, the molecular weight of 5′−NT isolated from *Escherichia coli* is 61 kDa [26]; that of 5′−NT isolated from black rockfish (*Sebastes inermis*) muscle is 67 kDa [8]; that of 5′−NT isolated from the venom of *Trimeresurus albolabris* is 48.03 kDa [27]; that of 5′−NT isolated from the venom of *Agkistrodon blomhoffii Ussurensis* is 70 kDa [25]; and that of 5′−NT isolated from cobra venom is only 10 kDa [29]. The commercial 5′−NT used in this experiment was extracted from microorganisms (2.6), while the purified 5′−NT was isolated from pork tenderloin. The difference in source may be the main reason for the difference in RP−HPLC retention time between the two enzymes [8,16].

#### 3.1.4. Molecular Weight Calculation

**Figure 4:**
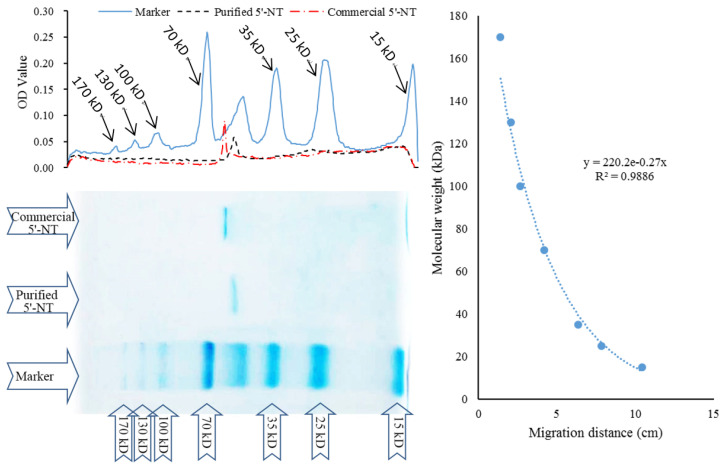
SDS−PAGE and its optical density analysis of the commercial and purified 5′−NT.

SDS−PAGE and the linear regression method were used to determine the molecular weight of the purified 5′−NT [24], and the results are shown in Figure 4. According to the regression equation, the calculated molecular weights of the purified and commercial 5′−NT were 57 kDa and 61.6 kDa, respectively. This result is consistent with that of the relative retention time of the compounds, which was shorter for the purified compound than for the commercial counterpart (see Section 3.1.3).

### 3.2. Enzymatic Properties

The enzymatic properties of endogenous 5′−NT represent an important basis for regulating its activity, the reduction of which might help to retain more umami−enhancing nucleotides in meat. Therefore, based on previous investigations on 5′−NT activity and methods used in meat processing [8,16], the main factors (temperature, pH, salt ions, and carbohydrate content) influencing 5′−NT activity were investigated.

#### 3.2.1. Optimal Temperature

**Figure 5:**
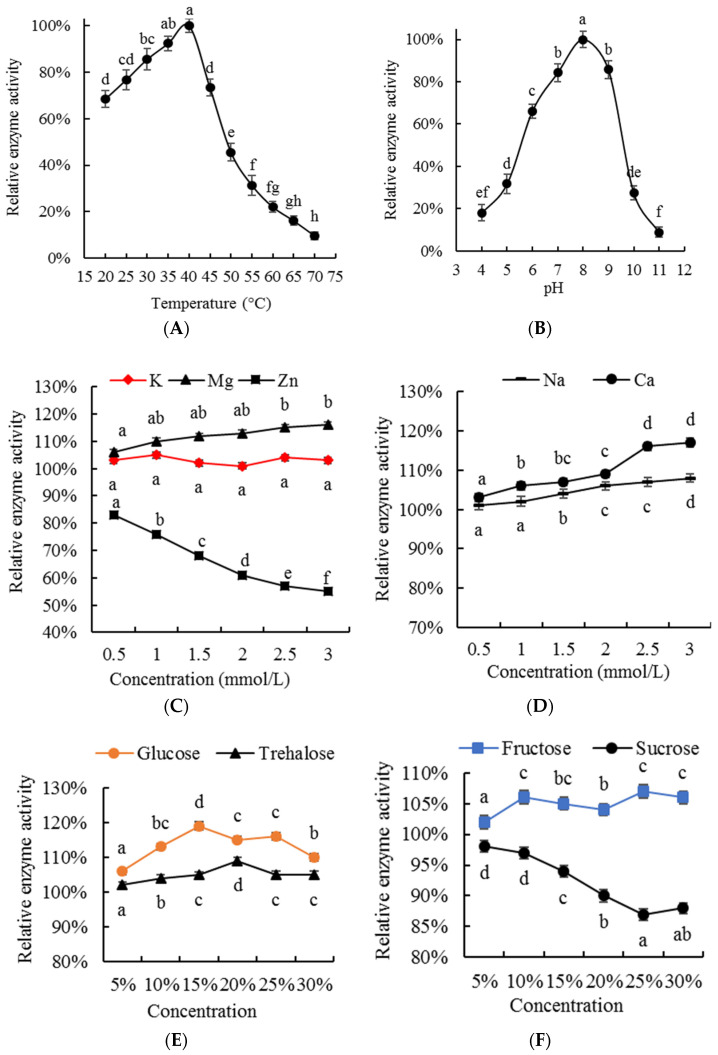
Enzymatic properties of the purified 5′−NT. Values are presented as mean ± standard deviation. Different letters above the bars or points highlighting the standard deviation indicate significant differences (*p* < 0.05). (**A**) Effect of temperature on activity of 5′−NT; (**B**) Effect of pH on activity of 5′−NT; (**C**) Effect of K^+^, Mg^2+^ and Zn^2+^ concentration on activity of 5′−NT; (**D**) Effect of K^+^, Na^+^ and Ca^2+^ concentration on activity of 5′−NT; (**E**) Effect of glucose and trehalose concentration on activity of 5′−NT; (**F**) Effect of fructose and sucrose concentration on activity of 5′−NT.

Temperature is one of the key factors affecting enzyme activity [30,31]. Low temperatures do not change the spatial structure of enzymes but interrupt their catalytic reactions; when the reaction temperature increases, enzyme activity can be restored. On the other hand, high temperatures can cause irreversible changes in the spatial structure of enzymes, permanently inhibiting enzyme activity [30,31]. The effect of reaction temperature on 5′−NT activity is shown in Figure 5A. The data in this figure indicate that the relative enzyme activity of 5′−NT increased with the increase in reaction temperature but decreased significantly (*p* < 0.05) above 40 °C. Therefore, the optimal reaction temperature for the purified 5′−NT was determined to be 40 °C. This result is similar to the optimal reaction temperature of 5′−NT extracted from rockfish (*Sebastes inermis*) muscle (45 °C) [8].

#### 3.2.2. Optimal pH

The reaction pH is another key factor affecting enzyme activity [30,31]. Due to the presence of many alkaline or acidic amino acid residues on the protein molecules of enzymes, changes in pH directly affect these side chains and residues by changing the enzyme’s substrate specificity, altering the enzyme’s spatial structure, exposing or hiding active sites, etc. [30]. Figure 5B shows the relative activity of the purified 5′−NT with different pH values. The data in this figure indicate that pH 8 is the optimal value for the purified 5′−NT. This result is consistent with the optimal pH values for purified 5′−NT from black rockfish (*Sebastes inermis*) muscle (8.3) [8] and *Bamboo Leaf Green Snake* venom (8.5–9.5) [16].

#### 3.2.3. Effect of Salt Ions on Purified 5′−NT Activity

Salt is frequently used in the preservation, water retention, and pickling of raw meat materials [32]. Exploring the effects of commonly used salt ions on 5′−NT activity is beneficial for selecting appropriate salt ions for meat processing and preservation, as they can inhibit or promote the degradation of umami−enhancing nucleotides in pork. Figure 5C,D show the effect of salt ions on the activity of the purified 5′−NT in this study. The results in these figures indicate that the relative activity of 5′−NT shows different changes with the increase in salt ion concentration: it significantly (*p* < 0.05) increased with the increase in the concentrations of Mg^2+^, Ca^2+^, and Na^+^; remained stable with K^+^ concentration variations, which had no significant (*p* > 0.05) effect; and significantly (*p* < 0.05) decreased with Zn^2+^ concentration variations. From these results, it can be inferred that Mg^2+^, Ca^2+^, and Na^+^ increase endogenous 5′−NT activity, K^+^ has no significant (*p* > 0.05) effect on it, and Zn^2+^ can inhibit it. Similar results have been reported in previous research on 5′−NT:Mg^2+^ and Ca^2+^ promoted the activity of 5′−NT derived from the venom of *Agkistrodon blomhoffii Ussurensis* [25]; Zn^2+^ showed an inhibiting effect (inhibiting ratio 97%) on the activity of 5′−NT from *Trimeresurus albolabris* venom [16]; K^+^ had a promoting effect on the activity of 5′−NT from the venom of *Agkistrodon blomhoffii Ussurensis* [25] but had no significant (*p* > 0.05) effect on the activity of purified 5′−NT. The different sources of enzymes and their protein structures may be the key factors leading to different effects of salt ions on 5′−NT activity.

#### 3.2.4. Effect of Carbohydrates on Purified 5′−NT Activity

Carbohydrates (fructose, glucose, sucrose, and trehalose) are commonly used meat additives [33], which can not only have seasoning−related effects but also function as anti−corrosion and preservation additives in meat processing and preservation. In order to evaluate the effects of different carbohydrates on 5′−NT activity, fructose, glucose, sucrose, and trehalose were considered (Figure 5D,E). The results in Figure 5D,E indicate that 5′−NT activity first significantly (*p* < 0.05) increased and then significantly (*p* < 0.05) decreased with the increase in glucose and showed a significant (*p* < 0.05) upward trend with the increase in trehalose and fructose, with peaks corresponding to amounts of trehalose and fructose of 20% and 10%, respectively. Sucrose, on the other hand, had an inhibitory effect on 5′−NT enzyme activity, with a significant decrease (*p* < 0.05) in activity being observed when the amount of sucrose was 15%; however, any further increase above 25% sucrose had no significant (*p* > 0.05) effects. According to these results, it can be inferred that the addition of fructose, glucose, and trehalose is beneficial for enhancing the activity of 5′−NT, while sucrose is beneficial for its inhibition.

### 3.3. Comparison of Hydrolysis Effect

**Figure 6:**
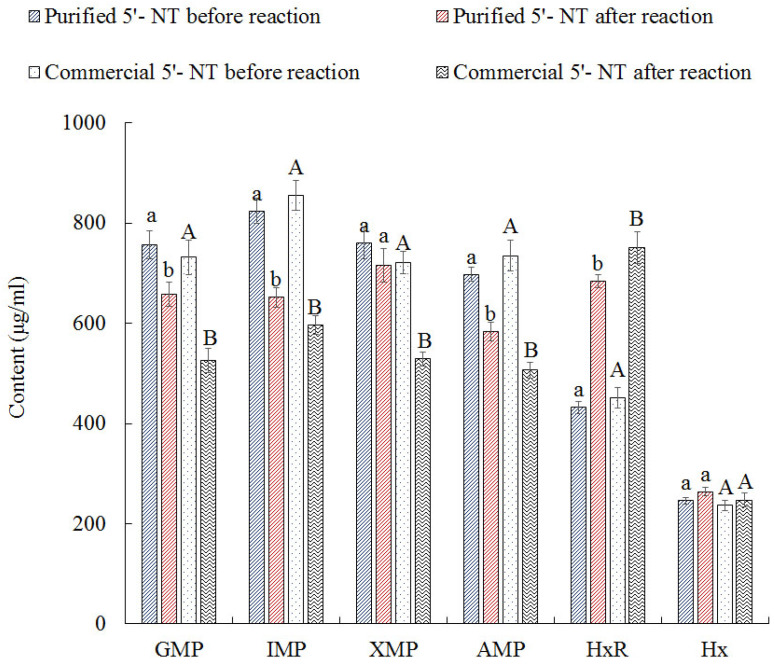
Substrate specificity of commercial and purified enzymes. Values are presented as mean ± standard deviation. Different letters above the bars or points highlighting the standard deviation indicate significant differences (*p* < 0.05).

To verify the hydrolysis effect of the purified 5′−NT, both the purified compound and its commercial counterpart were used to hydrolyze nucleotides (GMP, IMP, XMP, AMP, HxR, and Hx), and the obtained results are shown in Figure 6. After hydrolysis, the contents of GMP, IMP, and AMP decreased significantly (*p* < 0.05), while the content of HxR increased significantly (*p* < 0.05), and the content of Hx showed no significant (*p* > 0.05) increase. The content of XMP did not decrease significantly (*p* > 0.05) when treated with the purified 5′−NT, but it did when treated with the commercial compound. These results suggest that the purified 5′−NT has weak hydrolytic activity towards XMP. After hydrolysis with the purified 5′−NT, the contents of GMP, IMP, XMP, and AMP decreased by 12.96%, 20.98%, 5.96%, and 16.34%, respectively; after hydrolysis with the commercial 5′−NT, they decreased by 28.27%, 30.35%, 26.63%, and 31.14%, respectively. These results indicate that the purified compound has a hydrolysis effect similar to that of the commercial one with two exceptions: its hydrolysis activity against IMP is higher, and that against XMP is lower than that of the commercial 5′−NT tested.

5′−NT is a specific phosphatase that catalyzes the hydrolysis of 5′ nucleotides, mainly acting on the dephosphorylation of IMP [34,35]. This could be the main reason for the decreases in IMP content in the purified and commercial 5′−NT groups being higher than the decreases in the content of other nucleotides. The highest IMP content decrease was recorded in the purified 5′−NT hydrolysis group (20.98%), while in the commercial 5′−NT hydrolysis group, the decrease in IMP content (30.35%) was second to that in AMP content (31.14%). From these results, it can be seen that the purified 5′−NT showed superior degradation specificity towards IMP than the commercial counterpart. According to the nucleotide metabolic pathway [36], the hydrolysis of IMP by 5′−NT produces HxR, which is further hydrolyzed to produce Hx. This may be the main reason for the contents of HxR and Hx being increased after hydrolysis by the purified 5′−NT and commercial 5′−NT (see Figure 6).

### 3.4. Metabolism of Nucleotides by 5′−NT in Chilled Pork

**Figure 7:**
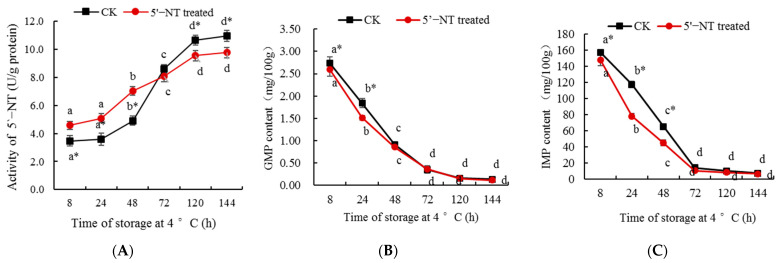

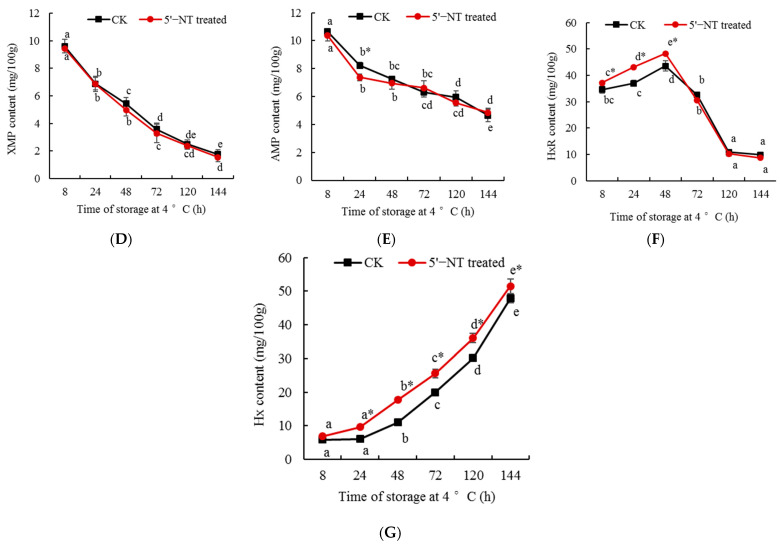
Effects of 5′−NT on the content of nucleotides of the pork stored at 4 °C. Values are presented as mean ± standard deviation. Different letters above the bars or points highlighting the standard deviation indicate significant differences (*p* < 0.05). “*” represents significant differences between groups (*p* < 0.05). (**A**) Effect of storage time on activity of 5′−NT; (**B**) Effect of storage time on content of GMP; (**C**) Effect of storage time on content of IMP; (**D**) Effect of storage time on content of XMP; (**E**) Effect of storage time on content of AMP; (**F**) Effect of storage time on content of HxR; (**G**) Effect of storage time on content of Hx.

In order to further verify the activity of the purified 5′−NT, the compound was added to chilled pork tenderloin samples, and its metabolic effect on nucleotides was determined (Figure 7). The results in Figure 7A show that, at 8 h, 24 h, and 48 h, 5′−NT activity in the treatment group was significantly (*p* < 0.05) higher than that in the control; at 72 h, there were no significant (*p* > 0.05) differences between the two groups; and at 120 h and 144 h, 5′−NT activity in the treatment group was significantly (*p* < 0.05) lower than that in the control. Figure 7B,C,E show that at 8 h, 24 h, and 48 h, the GMP, IMP, and AMP contents in the 5′−NT−treated group were lower than those in the control, while at 72 h, 120 h, and 144 h, there were no significant (*p* > 0.05) differences in their contents between the two groups. Figure 7D indicates that there were no significant (*p* > 0.05) differences in the content of XMP between the two groups. Figure 7F demonstrates that at 8 h, 24 h, 48 h, 72 h, 120 h, and 144 h, the content of Hx in the 5′−NT−treated group was higher than that in the control. Figure 7G indicates that at 8 h, 24 h, and 48 h, the content of HxR in the 5′−NT−treated group was higher than that in the control, but at 72 h, 120 h, and 144 h, there were no significant (*p* > 0.05) differences between the two groups. From these results, it can be inferred that the purified 5′−NT has a metabolic effect on the nucleotides in pork tenderloin under −4 °C storage conditions. The preparation of 5′−NT enhances our understanding of its enzyme activity, laying a solid foundation for regulating the metabolism of nucleotides in pork through the control of the factors that affect the activity of this compound.

The treatment group included samples supplemented with 5′−NT and allowed to stand, which may be the reason why 5′−NT activity in the treatment group was higher than that in the control at 8 h, 24 h, and 48 h (Figure 7A). On the contrary, 5′−NT activity in the treatment group was lower than that in the control at 120 h and 144 h (Figure 7A), which indicates that with the increase in storage time, the high content of 5′−NT accelerated the metabolism of nucleotides in the meat, leading to a large depletion in 5′−NT. The contents of GMP, IMP, and AMP in the 5′−NT−treated group were lower than those in the control (Figure 7B,C,E), indicating that the high content of 5′−NT in the treatment group at 8 h, 24 h, and 48 h accelerated the metabolism of these compounds. The content of XMP showed no significant (*p* > 0.05) differences between the 5′−NT−treated and control groups at 8 h, 24 h, 48 h, 72 h, 120 h, and 144 h (Figure 7D), indicating that the degradation activity of 5′−NT against XMP was not high (Figure 6). According to nucleotide metabolic pathway [36], after having been degraded by 5′−NT, IMP produces HxR, which is further hydrolyzed to produce Hx. At 8 h, 24 h, and 48 h, 5′−NT activity in the treatment group was significantly (*p* < 0.05) higher than that in the control, resulting in more IMP being converted into HxR, thus further resulting in the content of HxR in the 5′−NT−treated group being significantly (*p* < 0.05) higher than in the control at 8 h, 24 h, and 48 h (Figure 7F). At 72 h, 120 h, and 144 h, there were no significant (*p* > 0.05) differences in the content of HxR between the 5′−NT−treated group and the control (Figure 7F), indicating that the degradation of HxR resulted in an accumulation of Hx in the 5′−NT−treated group, which led to the content of Hx in the 5′−NT−treated group being higher than that in the control at 24 h, 48 h, 72 h, 120 h, and 144 h (Figure 7G).

## 4. Conclusions

The molecular weight, optimal temperature, and optimal pH of the purified 5′−NT obtained in this study were 57 kDa, 40 ° C, and 8, respectively. Zn^2+^ and sucrose inhibited the activity of the purified 5′−NT, while K^+^, Na^+^, Ca^2+^, Mg^2+^, glucose, fructose, and trehalose promoted it. The purified compound had higher catalytic activity against IMP than commercial 5′−NT but showed no significant (*p* > 0.05) catalytic activity against XMP. The purified 5′−NT was further used in chilled pork tenderloin, and similar results were obtained in the degradation of GMP, IMP, XMP, AMP, Hx, and HxR standards. In brief, we successfully isolated 5′−NT from pork tenderloin by using a novel methodology, which enhanced our understanding of its enzyme activity and provided a theoretical basis for regulating nucleotide metabolism in pork through the control of endogenous 5′−NT activity. However, the extraction processing needs further optimization. Therefore, in the future, we will focus on (1) optimizing the extraction processing with the response surface method, making the extraction processing suitable for large−scale extraction, and (2) regulating nucleotide metabolism in chilled pork by adding a given mass of Zn^2+^ and sucrose.

## Figures and Tables

**Table 1:** Summary of purification of 5′−NT from carp muscle.

Fractions	Total Protein (g/L)	Total Activity (U/L)	Specific Activity (U/g Protein)
Sephadex Fraction I (F−I)	2.67 ± 0.09 ^a^	18.35 ± 0.33 ^a^	6.87 ± 0.28 ^a^
Sephadex Fraction II (F−II)	1.98 ± 0.05 ^b^	3.82 ± 0.15 ^b^	1.92 ± 0.06 ^b^
RP−HPLC F−I−1	0.86 ± 0.06 ^A^	35.42 ± 0.87 ^A^	41.19 ± 1.98 ^A^
RP−HPLC F−I−2	0.23 ± 0.03 ^B^	1.24 ± 0.06 ^B^	5.39 ± 0.28 ^B^
RP−HPLC F−I−3	0.29 ± 0.04 ^B^	1.51 ± 0.02 ^B^	5.21 ± 0.32 ^B^

Values are presented as mean ± standard deviation. Different letters beside the standard indicate significant differences (*p* < 0.05).

## Data Availability

Data are contained within the article.

## References

[1] Zhang Y., Zhang Y., Jia J., Peng H., Qian Q., Pan Z., Liu D. (2023). Nitrite and nitrate in meat processing: Functions and alternatives. Curr. Res. Food Sci..

[2] Zhang Y., Zhang Y., Li H., Bai T., Qian Q., Peng H., Mu Y., Wang L., Liu B., Chen J. (2023). Effect of 4 °C and ice temperature on umami-enhancing nucleotides of conditioned pork. Food Chem..

[3] Zhang Y., Zhang Y., Li H., Guo T., Jia J., Zhang P., Wang L., Xia N., Qian Q., Peng H. (2022). Comparison of Nutrition and Flavor Characteristics of Five Breeds of Pork in China. Curr. Res. Food Sci..

[4] Zhang Y., Venkitasamy C., Pan Z., Liu W., Zhao L. (2017). Novel Umami Ingredients: Umami Peptides and Their Taste. J. Food Sci..

[5] Zhang Y., Zhang L., Venkitasamy C., Pan Z., Ke H., Guo S., Wu D., Wu W., Zhao L. (2020). Potential effects of umami ingredients on human health: Pros and cons. J. Food Sci..

[6] Zhang Y., Li H., Zhang Y., Wang L., Zhang P., Jia J., Qian Q., Zhang J., Pan Z., Liu D. (2022). Storage Stability and Flavor Change of Marinated Pork. Curr. Res. Food Sci..

[7] Zhang Y., Venkitasamy C., Pan Z., Wang W. (2013). Recent developments on umami ingredients of edible mushrooms—A review. Trends Food Sci. Technol..

[8] Marseno D.W., Hori K., Miyazawa K. (1993). Purification and properties of membrane-bound 5′-nucleotidase from black rockfish (Sebastes inermis) muscle. J. Agric. Food Chem..

[9] Fernández-Justel D., Peláez R., Revuelta J.L., Buey R.M. (2019). The Bateman domain of IMP dehydrogenase is a binding target for dinucleoside polyphosphates. J. Biol. Chem..

[10] Burger R.M., Lowenstein J.M. (1970). Preparation and Properties of 5′-Nucleotidase from Smooth Muscle of Small Intestine. J. Biol. Chem..

[11] Evans W.H., Gurd J.W. (1973). Properties of a 5′-nucleotidase purified from mouse liver plasma membranes. Biochem. J..

[12] Dornand J., Bonnafous J.C., Mani J.C. (1978). Purification and Properties of 5′-Nucleotidase from Lymphocyte Plasma Membranes. Eur. J. Biochem..

[13] Song C.S., Bodansky O. (1966). Purification of 5′-Nucleotidase from Human Liver. Biochem. J..

[14] Yamamoto H., Tomioka K., Kawai H., Endo K. (1986). Purification and Properties of 5′-Nucleotidase from Cod Muscle. Agric. Biol. Chem..

[15] Tomioka K., Endo K. (1984). Purification of 5′-nucleotidase from carp muscle. Bull. Jpn. Soc. Sci. Fish..

[16] Chen X. (2008). Study on 5′-Nucleotidase from *Trimeresurus Albolabris* Venom of Its Purification, Physicochemical Property and Mechanism of Inhibiting Rabbit Platelet Aggregation. Master’s Thesis.

[17] Montero J.M., Fes J.B. (1982). Purification and characterization of bovine brain 5′-nucleotidase. J. Neurochem..

[18] Neu C.H. (1967). The 5′-Nucleotidase of *Escherichia coli*: I. Purification and Properties. J. Biol. Chem..

[19] Lin S., Zhou C., Zhang H., Cai Z. (2019). Expression, purification and characterization of 5′-nucleotidase from caterpillar fungus by efficient genome-mining. Protein Expr. Purif..

[20] Yusupova Y.R., Skripnikova V.S., Kivero A.D., Zakataeva N.P. (2020). Expression and purification of the 5′-nucleotidase YitU from Bacillus species: Its enzymatic properties and possible applications in biotechnology. Appl. Microbiol. Biotechnol..

[21] Jin H.G., Wan K.H., Tian R.H., Peng Z.Q., Rong W.R. (2012). Purification and Characterization of Pyrophosphatase from Pork longissimus dorsi Muscle. Food Sci..

[22] Yao Z.L. (2012). Purification and Some Characterization of a Group of Glycolysis Enzymes and Alkaline Phosphatase from Pig Muscle Tissue. Master’s Thesis.

[23] Zhang Y., Zhu Z.W., Zeng Q.X. (2011). Effect of ultrasonic treatment on the activities of endogenous transglutaminase and proteinases in tilapia (*sarotherodon nilotica*) surimi during gel formation. J. Food Process Eng..

[24] Zhang Y., Hui W.X., Wang W., Lu X.F., Hu J.Y. (2015). Electrophoretic Separation of Proteins and Peptides in Bone Extract and Bone ExtractHydrolysates. Mod. Food Sci. Technol..

[25] Zhang B.Y. (2010). Purification and Characterization of 5′-Nucleotidase from *Agkistrodon blomhoffii Ussurensis* Snake Venom. Master’s Thesis.

[26] Allegrini S., Pesi R., Tozzi M.G., Fiol J.C., Johnson B.R., Eriksson S. (1997). Bovine cytosolic IMP/GMP-specific 5′-nucleotidase: Cloning and expression of active enzyme in *Escherichia coli*. Biochem. J..

[27] Chen X., Xiao-Dong Y.U., Deng M., Li H., Liu J.P. (2008). Purification and Characterization of 5′-nucleotidase from Trimeresurus albolabris Venom. Zool. Res..

[28] Ovissipour M., Rasco B., Shiroodi S.G., Modanlow M., Gholami S., Nemati M. (2013). Antioxidant activity of protein hydrolysates from whole anchovy sprat (*Clupeonella engrauliformis*) prepared using endogenous enzymes and commercial proteases. J. Sci. Food Agric..

[29] Yu C.G., Ren Y.Z. (1987). Purification of 5′-nucleotidase from snake venom of Naja Naja Atra. J. China Med. Univ..

[30] Suzuki H. (2019). How Enzymes Work: From Structure to Function.

[31] Yan S. X., Cai H.Y. (1987). Principles and Methods of Enzyme Catalytic Kinetics.

[32] Zhou G.H. (2002). Animal Product Processing.

[33] Hu G. (2005). The Application of Food Additives in Poultry, Livestock and Aquatic Products.

[34] Morgan M.J. (1996). Studies on 5′-nucleotidase N-I..

[35] Tang L., Yang D., Wang Y., Yang X., Chen K., Luo X., Xu J., Liu Y., Tang Z., Zhang Q. (2021). 5′-Nucleotidase Plays a Key Role in Uric Acid Metabolism of *Bombyx mori*. Cells.

[36] Chandel N.S. (2021). Nucleotide metabolism. Cold Spring Harb. Perspect. Biol..

